# The Educative Work of the School Doctor

**Published:** 1915-07-03

**Authors:** 


### THE EDUCATIVE WORK OF THE SCHOOL DOCTOR.*

### II.—Lantern Slides for Parents' Lectures.

By A LATELY RETIRED SCHOOL DOCTOR.

Lantern-slides greatly help in giving lectures to parents. In the first place they provide admirable illustrations for points that can only be explained in a circumlocutory way. Secondly, they give the audience a trifle of breathing-time in which to absorb the information given them. Thirdly, most audiences like them if they are interesting.

Unfortunately, lantern-slides are not easily obtainable. I have been at some pains to obtain a decent set of slides for parents' meetings in England, and have been struck by the uselessness of the various slides listed by makers in London and the provinces. Undoubtedly the most "taking" slides are those with some local interest. If you are a photographer yourself and take an interest in such things you can easily make them yourself. A collection of slides illustrating life in the schools under your charge will have an enduring value and will always be of great service. But those of us who are not expert photographers cannot depend on such self-help; we have to go elsewhere. Sometimes a teacher will be found who has taken up photography as a hobby and who has made excellent lantern-slides. Such assistance will be most useful, for the enthusiast can be turned on subjects of medico-social interest and set to work to provide a set of slides for school meetings. Some of the best slides in my collection, unique in their interest, are snapshots taken by enthusiastic teachers. The range of subjects for such slides is immense. First, there is the routine of school inspection. It is, of course, now pretty familiar to all parents, but they like to see it illustrated on the screen, and a yearly lecture on school inspection itself is always useful. There are still parents who think it useless cruelty to strip a child as a preliminary to inspection; "it catches its death of cold, the poor thing," they complain. A five minutes' chat on the elements of auscultation and percussion, I find, usually makes such recalcitrant parents willing assistants at the next inspection.

In the course of several years' inspection I have collected some six hundred lantern-slides illustrating various matters of school and medico-social interest. From these it is easy to pick out three or four dozen to illustrate an afternoon's lecture on some special subject. More than fifty slides for a lecture are undesirable; the pictures are too crowded and the time allowed for their due explanation and discussion, and, be it added, appreciation, is too short. A parents' lecture should never exceed an hour, and a good lantern picture increases in interest the more it is inspected, since unsuspected points of interest are overlooked at the first glance. So do not be too prodigal with your slides. It is, of course, not easy to collect such a number of good slides, especially under the limitations which one has to set. These are that the slides should be of popular interest, strongly and lastingly made, perfectly clear, and of such a nature that they need little explanation. Every slide should tell its own story. For lectures to teachers and for occasional illustrations to papers of a technical nature, slides of a more special kind are needed, and the collection should contain some dozen of these. Most good makers supply slides, but, as I have already stated, in England no special effort has been made to cater for the school doctor's needs. German firms used to supply excellent slides, while French, Belgian, and Dutch makers often list out-of-the-way pictures that are very useful. But far and away the best slides for use at such lectures are those sold by Ciglia, of Genoa (Via G. Ferrari 6), who is the agent for the "Federazione Italiana delle opere antitubercolari." This firm's catalogue lists thousands of first-class slides on every conceivable subject in connection with the campaign against tuberculosis. The slides may be had in negative colours or in colour, and they are all listed at a uniform price of one lira, or ninepence each, uncoloured. I have found the make and quality of these Italian lantern-slides far superior to anything manufactured elsewhere, and the choice in the catalogue is so extensive that a school doctor will find all his requirements met. A special feature of this firm is the excellent series of slides illustrating open-air schools; the pictures are not taken from Italian schools alone, but from schools all over the world, and practically every open-air school in existence is represented. Another feature is the admirable series of statistical slides showing the economic aspects of preventable diseases; some of these are highly graphic, and should be understood by every audience; a few are suitable only for Italian audiences, but with some ingenuity the school doctor can easily make similar slides by copying on an exposed lantern slide plate, using aniline ink for the purpose, the statistical table given, and translating the lettering. Yet a third feature is the really useful series representing the historical aspect of tuberculosis. Most of the slides listed in the catalogue are popular slides, but several series are highly technical, and are suitable for lectures to students. I may mention the series illustrating bacteria, cutaneous diseases, and the pathology of tuberculosis. Among the last mentioned are most beautiful slides, showing tuberculous lesions of the vertebræ causing compression of the medulla, double perforation of the pleura with pneumothorax, caseous peribronchitis, and tuberculous synovitis of the metatarsal joints.

Most schools are provided with a proper lantern, which one of the teachers manipulates. In halls and in country districts the doctor may have to bring his own lantern. The most useful is undoubtedly a plug electrically-lighted lantern, but its usefulness is limited to places where electric light is

* Article I. appeared on June 19.

laid on. A simple and convenient apparatus is that in which acetylene is used as the lighting medium. Acetylene lanterns are listed by all firms that supply such instruments and apparatus; they are cheap, and stand the wear and tear of travelling very well.

Provided with a set of lantern-slides, and with the ability to explain simple things in simple language, the school doctor increases his utility a hundred-fold. Probably he similarly increases his popularity, but at all events he has the satisfaction of knowing that he is doing a highly acceptable service to the community in undertaking such educative work. The beneficent results of a year's lecturing on such subjects as tuberculosis, adenoids and tonsils, rickets, infectious diseases, elementary hygiene, care of the teeth, clothing, cleanliness of the head and the body, simple cookery, emergencies, the common cold, the ventilation of the home and the living and sleeping room, the care of the baby and of the school-going child, will be easily apparent when revision time comes round. Numerous interesting subjects can be found, and if discussion is encouraged many parents will specifically ask for some particular subject to be dealt with. Thus I have been asked to "talk" on "underclothing," "Whether two children should sleep in the same bed," "If it's bad for children to take beer," and "What to do with fractious children"—all, it will be seen, highly interesting subjects that can be made the nucleus for an all-round crystallisation of the elementary facts of child and home hygiene.

